# Controlled flow reduction of an iliacoportal shunt graft for portal vein arterialization in a pediatric patient

**DOI:** 10.1007/s00247-023-05733-3

**Published:** 2023-08-24

**Authors:** Charlotte Kulka, Susanne Lagrèze, Niklas Verloh, Michael Doppler, Simone Hettmer, Stefan Fichtner-Feigl, Wibke Uller

**Affiliations:** 1 Department of Diagnostic and Interventional Radiology, Medical Center University of Freiburg, Faculty of Medicine, University of Freiburg, Hugstetter Straße 55, 79106 Freiburg, Germany; 2 Department of General and Visceral Surgery, Medical Center Freiburg, University of Freiburg, Hugstetter Strasse 55, Freiburg, Germany; 3 Department of Pediatrics and Adolescent Medicine, Division of Pediatric Hematology and Oncology, Faculty of Medicine, University of Freiburg, Freiburg, Germany

**Keywords:** Interventional radiology, Liver, Pediatric, Portal hypertension, Portal vein arterialization, Stent graft

## Abstract

Portal vein arterialization is a rarely used, temporary surgical salvage solution to prevent biliary and hepatic ischemia and necrosis in acute liver de-arterialization. However, it can induce portal hypertension, causing increased morbidity and mortality. We report the case of a 5-year-old girl with portal hypertension and right ventricle volume overload following the creation of an iliacoportal shunt graft for portal vein arterialization due to vessel-adhering neuroblastoma. Partial shunt graft closure was accomplished by placing a stent graft in an hourglass configuration via the right femoral artery using two slender-sheaths in a line with the second more distal than the first. Subsequently, the patient’s symptoms of right ventricle volume overload and portal hypertension decreased. In conclusion, endovascular reduction of elevated portal blood flow after portal vein arterialization is feasible, even in pediatric patients.

**Graphical abstract:**

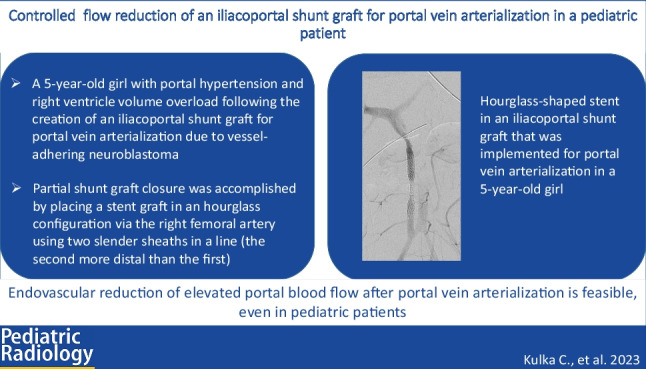

## Introduction

Portal vein arterialization represents a rarely performed surgical salvage technique aiming to establish hepatic and biliary blood flow [1, 2]. The hepatic arteries are the main oxygen supplier to the biliary tract and also supply a quarter of the blood carried to the liver; thus, portal vein arterialization serves to avoid biliary and liver ischemia and necrosis [1, 2]. Indications for portal vein arterialization are acute de-arterialization of the liver (hepatic artery injury or obstruction) and failure to reconstruct the hepatic artery [2]. Portal vein arterialization increases blood flow in the portal vein and may cause portal hypertension [1]. While portal vein arterialization is only a temporary solution to bridge acute de-arterialization and the development of arterial collateralization, the consequences of portal hypertension may be longstanding, specifically alteration of the liver parenchyma and liver fibrosis [3]. We present the case of a pediatric patient who underwent portal vein arterialization and endovascular flow reduction of an arterial-portovenous shunt graft.

## Description

**Fig. 1:**
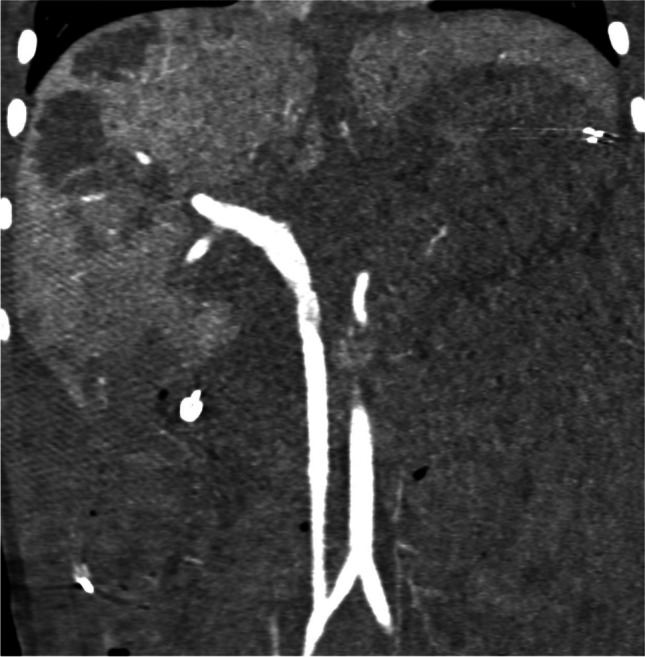
Coronal contrast-enhanced computed tomography image in a 5-year-old girl reveals contrast within a shunt graft between the right iliac artery and the portal vein to achieve portal vein arterialization in an otherwise de-arterialized liver

A 5-year-old girl (17 kg bodyweight) was diagnosed with recurring neuroblastoma (MYCN non-amplified, intermediate risk). The neuroblastoma was initially diagnosed in utero and her previous treatment regimen included chemotherapy and subtotal tumor resection. On follow-up magnetic resonance imaging, the tumor measured 3.9 × 2.3 × 4.9 cm and adhered to the celiac trunk, the common hepatic artery, the portal vein and the superior mesenteric artery, as well as the right renal artery and vein. Total tumor resection was performed, sparing the portal vein but including resection of the hepatic artery due to tumor adherence. Due to the vast tumorous vascular involvement, end-to-side anastomosis of the celiac trunk or the splenic artery with the portal vein was technically impossible. Hence a team of visceral surgeons (including S.F-F. with 25 years of experience in visceral surgery and S.L., specialized in pediatric surgery for 23.5 years) interposed a polytetrafluoroethylene graft (PTFE, 6 mm diameter, 10 cm length, Impra Carboflo, Becton, Dickinson and Company, Franklin Lakes, NJ) between the right common iliac artery and portal vein to achieve portal vein arterialization (Fig. 1). Because ultrasound and contrast-enhanced computed tomography performed 9 h after the surgery confirmed graft-thrombosis, surgical thrombectomy of the vascular graft and the portal vein was conducted by a team of visceral and vascular surgeons to re-establish hepatic blood flow. Afterwards, the patient was given unfractionated heparin in therapeutic doses. The diagnosis of protein S deficiency was established and further investigation revealed that the protein S deficiency was acquired postoperatively. On the fifth postoperative day, Doppler ultrasound showed an increased hepatopetal flow of the portal vein at the liver hilum of 60–80 cm/s (in adults, normal portal vein peak systolic velocity ranges around 20–40 cm/s; to our knowledge, there are no defined pediatric reference values). Furthermore, the patient suffered from ascites, osmotic diarrhea due to hyperperfusion of the intestine and right ventricular volume overload; hence, the diagnosis of portal hypertension was established. After interdisciplinary discussion, an endovascular partial closure of the shunt graft from 6 mm to 3 mm was scheduled 29 days after the creation of the shunt graft.

**Fig. 2:**
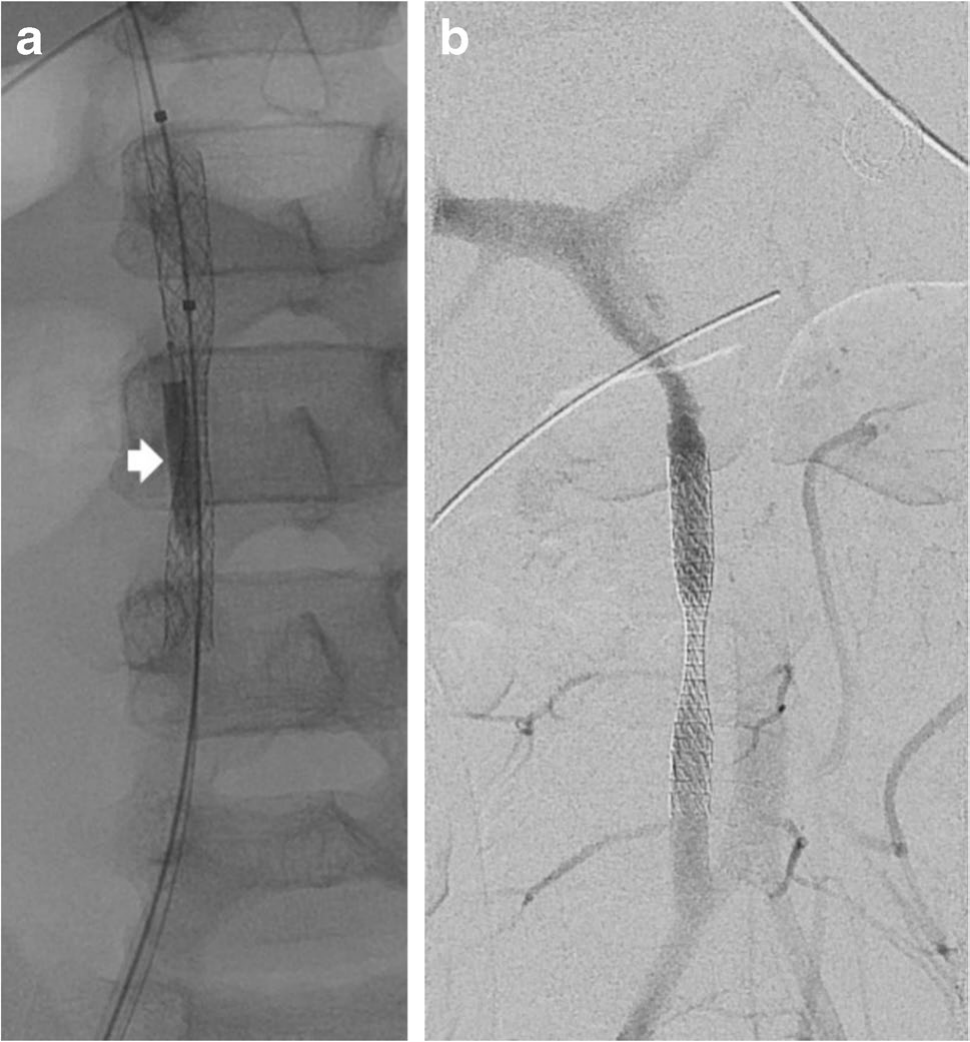
A 5-year old girl with neuroblastoma and an iliacoportal shunt graft for portal vein arterialization. **a** Posteroanterior (PA) fluoroscopy image shows a deployed stent within the iliacoportal shunt graft with an inflated angioplasty balloon (*arrow*) positioned outside of the stent resulting in an hourglass configuration of the stent. **b** PA digital subtraction angiography image after stent deployment demonstrates an hourglass-shaped stent graft placed within the iliacoportal shunt graft to reduce the shunt graft diameter and blood flow to the portal vein

The procedure was performed under general anesthesia in an angiography suite by an interventional radiologist specialized in pediatric interventions (W.U. with 14 years of experience in interventional radiology and specialized in pediatric interventional radiology for 12 years). The right common femoral artery was punctured under ultrasound guidance and via a 6-F slender-sheath (6F-Glidesheath slender, Terumo, Tokyo, Japan), the shunt graft and the portal vein were accessed (4F-RIM, Cordis, Miami Lakes, FL; 0.035" Glidewire, Terumo, Tokyo, Japan). The initial digital subtraction angiography (DSA) showed absence of any hepatopetal collaterals. After puncturing the left common femoral artery, multiple attempts to access the vascular graft from crossover remained unsuccessful due to the challenging anatomy. Consequently, the right common femoral artery was again punctured distal to the first puncture site and the tip of a 4-F slender-sheath (4F-Glidesheath slender, Terumo) was placed distal to the 6-F slender sheath to avoid occlusion of the femoral artery by parallel placement. Using the 6-F sheath, a stent graft (7 × 57 mm BeGraft, Bentley, Hechingen, Germany) was placed in the middle of the shunt graft and via the 4-F sheath, an angioplasty balloon (3.5 × 20 mm TREK, Abbot, Abbot Park, IL) was positioned in parallel and inflated before stent deployment (Fig. 2). Following the stent graft deployment, the distal and proximal portions of the stent were flared using a 7-mm balloon (7 × 20 mm EverCross, Medtronic, Minneapolis, MN) to create an hourglass configuration. Afterward, both angioplasty balloons were withdrawn. Completion DSA demonstrated the stent graft with a central narrowing measuring 3 mm and preserved contrast flow within the stent and the portal vein (Fig. 2). Post-interventional Doppler ultrasound showed a reduced hepatopetal flow of the portal vein of 40 cm/s and an open vascular graft. The patient’s ascites, diarrhea and right ventricular volume overload decreased in the days following the stent placement. Contrast-enhanced computed tomography performed 18 days after the stent graft implantation (46 days after portal vein arterialization) revealed occlusion of the ilioportal shunt graft and preserved portal vein blood flow. The right and the left hepatic artery were contrasted again due to blood supplied by arterial collaterals and both common femoral arteries were patent. At follow up six months after the stent graft implantation, the patient remained alive, free from any biliary or hepatic complications.

## Discussion

Portal vein arterialization has been described as a rescue option when hepatic artery reconstruction is not possible for oncologic or technical reasons in adults [1, 2] and in children with liver transplantation [4]. The majority of the hepatic blood supply, approximately 75%, originates from the portal vein, while the remaining blood supply is provided by the hepatic arteries, which are also the main oxygen suppliers of the biliary tract [2]. The primary goal of portal vein arterialization is to avoid biliary and liver ischemia and necrosis and, therefore, liver failure and death [1]. In the literature, different surgical techniques to perform a portal vein arterialization are described, generally favoring medium-sized and smaller arteries as well as end-arteries to reduce the risk of portal hypertension [1]. In our case, the neuroblastoma adhered to multiple visceral arteries, amongst them the superior mesenteric artery, celiac trunk, and splenic artery rendering their branches for an anastomosis or a jump graft to the portal vein technically impossible. The salvage portal vein arterialization was thus performed by creating an iliacoportal shunt graft using a vascular PTFE graft. On the one hand, the need to interpose a 6-mm PFTE graft between the iliac artery and the portal vein led to the patient’s increased portal flow, consecutively to portal hypertension and a right ventricle volume overload. On the other hand, due to the patient’s protein S deficiency, early thrombosis of the shunt graft occurred even using this diameter. Since both thrombosis and portal hypertension are known to represent the most common complications after portal vein arterialization, vessel or shunt graft caliber adjustment is quite tricky.

To date, there is no data available about minimal invasive endovascular therapy for the treatment of portal hypertension after portal vein arterialization—neither in pediatric nor in adult patients. Transjugular intrahepatic portosystemic shunt (TIPS) establishes an anastomosis by placing a stent graft between a hepatic vein and a portal vein branch to reduce portal hypertension. Creating a TIPS can lead to hepatic encephalopathy, necessitating a reduction in the diameter of the stent. There are different minimally invasive techniques for TIPS flow reduction. One technique was described in a case report by Holden et al., where they successfully created an hourglass shape in the original TIPS tract by using a second covered stent graft (Viatorr, W.L. Gore & Associates, Flagstaff, AZ). This was achieved by simultaneously inflating and deploying a balloon expandable stent (Palmaz Genesis; Cordis, Miami Lakes, FL) outside of the covered stent graft [5]. A significant reduction of the symptoms of the patient’s encephalopathy after the stent placement was reported [5]. Holden et al. used 6-F and 10-F sheaths implemented in two different puncture sites in the right internal jugular vein [5]. In our case, a modified technique was used since we required two small arterial accesses in a 5-year old child. Our experience demonstrated that (1) this technique is also feasible in young children by using two sheaths in a line with the second more distal than the first and (2) that this technique is able to partially occlude an arterial PFTE shunt graft used for portal vein arterialization with innovative stent grafts requiring small sheath diameters.

There are no recommendations for sheath size in the pediatric population; however, studies have shown that a larger sheath size proportionally increases the risk of puncture-site-associated complications [6]. We used slender sheaths (Glidesheath Slender, Terumo) with a smaller outer diameter than that of standard sheaths. After numerous attempts at entering the vascular graft from crossover remained unsuccessful, the right common femoral artery was punctured twice more, distally. A transfemoral parallel sheath technique has been previously described in 90 adults undergoing the treatment of chronic coronary occlusions, showing that this technique is feasible and safe in most patients [7]. We used two sheaths in a line with the second more distal than the first and showed that this approach is technically feasible in a pediatric patient with no access-related complications. This technique may be an alternative to using a single large caliber sheath or two parallel sheaths when two guide wires are necessary; however, more studies in the adult and pediatric population are required to validate the technique.

Animal studies showed that long-term overarterialization of the portal vein could lead to liver parenchymal changes and fibrosis [3]. Portal vein arterialization is thus not a long-term solution but serves as a temporary solution until arterial hepatopetal collaterals develop. The hepatopetal collaterals form due to the enlargement of preexisting arteries [2]. Some authors recommend reevaluating arterial collaterals 4–6 weeks after the surgery and using angiography to occlude the portal vein arterialization [8]. While it is also recommended to close the portal vein arterialization completely if portal hypertension occurs postoperatively [8], no long time studies support this and the benefit and ideal time for portal vein arterialization closure remain uncertain [2]. We favored partial closure to maximize time for arterial collaterals to develop and thus avoid biliary and liver necrosis. Interestingly, signs of portal hypertension and cardiac overload improved after partial shunt graft closure. After achieving sufficient arterial revascularization of the liver, the shunt graft demonstrated complete spontaneous occlusion 46 days following its creation. However, timely closure of the portal vein arterialization should always be considered once arterial collaterals are observed, to reduce the risk of complications caused by portal vein arterialization during follow-up.

## Conclusion

In conclusion, portal hypertension and right ventricle volume overload resulting from portal vein arterialization can be treated with minimal invasion by implementing an hourglass-shaped stent graft in the vascular graft, even in pediatric patients. This intervention may reduce portal hypertension and right ventricle volume overload, thus decreasing morbidity during the bridging period of hepatopetal collateral formation.

## Data Availability

Not applicable.
